# Terpenes as Green Solvents for Extraction of Oil from Microalgae

**DOI:** 10.3390/molecules17078196

**Published:** 2012-07-09

**Authors:** Celine Dejoye Tanzi, Maryline Abert Vian, Christian Ginies, Mohamed Elmaataoui, Farid Chemat

**Affiliations:** Université d’Avignon et des Pays du Vaucluse, INRA, UMR408, UMR406, 84000 Avignon, France; Email: celine.dejoye@univ-avignon.fr (C.D.T.); christian.ginies@avignon.inra.fr (C.G.); mohamed.elmaataoui@univ-avignon.fr (M.E.); farid.chemat@univ-avignon.fr (F.C.)

**Keywords:** lipid extraction, terpene solvent, *Chlorella vulgaris*, Soxhlet, microalgae

## Abstract

Herein is described a green and original alternative procedure for the extraction of oil from microalgae. Extractions were carried out using terpenes obtained from renewable feedstocks as alternative solvents instead of hazardous petroleum solvents such as *n*-hexane. The described method is achieved in two steps using Soxhlet extraction followed by the elimination of the solvent from the medium using Clevenger distillation in the second step. Oils extracted from microalgae were compared in terms of qualitative and quantitative determination. No significant difference was obtained between each extract, allowing us to conclude that the proposed method is green, clean and efficient.

## 1. Introduction

Extraction of oils from microalgae is a much more difficult problem than extraction of oils from oilseeds. Microalgae are single-cell organisms with extremely tough cell walls that can be difficult to disrupt [1]. The most common techniques for lipid extraction from microalgae in current use involve chloroform/methanol mixtures or hexane in solvent or Soxhlet extraction. These flammable and toxic organic solvents cause adverse health and environmental effects.

Currently, *n*-hexane, derived from petroleum, is the solvent of choice for extraction of oils. Its chemical properties provide ideal functionality as an extraction solvent for oils. *n*-Hexane is a light paraffinic petroleum fraction, has a fairly narrow boiling point range of 69 °C and is an excellent solvent in terms of oil solubility and ease of recovery. However, this solvent can be emitted during extraction and recovery and has been identified as an air pollutant since it can react with other pollutants to produce ozone and photochemical oxidants [2,3]. Safety, environmental and health concerns have increased the interest in alternatives to *n*-hexane in order to reduce emissions of volatile organic compounds into the atmosphere. Due to the new emphasis on environmental protection and the development of green chemistry, such solvent use is to be avoided as much as possible. The green chemistry is based on twelve principles [4] such as “design less hazardous chemical syntheses” (use substances with little or no toxicity to humans and the environment) or “use safer solvents and reaction conditions” (avoid using solvents). The twelve principles provide a good basis for researchers who want to develop new, more environmentally acceptable experiments. The most feasible alternative to *n*-hexane as solvent for extraction seems to be the replacement of this solvent by bio-solvents such as terpenes which as recognized as environmentally safer. In this study *d*-limonene, α-pinene and *para*-cymene were used in order to replace *n*-hexane.

Terpenes are natural solvents existing both in citrus fruits and in many other plants, with extraordinary technical and chemical properties. They include hydrocarbons with C_5_H_8_ isoprene units and are derivable chiefly from essential oils, resins, and other vegetable aromatic products. Many terpenes are acyclic, bicyclic, or monocyclic, and differ somewhat in physical properties. They represent an optimal alternative to petroleum solvents in many industrial applications. *d*-Limonene is a low cost, low toxicity biodegradable terpene present in agricultural wastes derived from citrus peels. As such this reagent can be considered as an economical renewable feedstock. It’s a very versatile chemical which can be used in a wide variety of applications. The growing interest of limonene has emerged since its cleaner and degreaser qualities were recognized and taken into consideration [5]. Recently, as an alternative to organic solvent extraction, the extraction of oil from oil-containing materials with *d*-limonene has been investigated [6,7,8]. The yield and quality of crude oil obtained from the *d*-limonene extraction were almost similar to those obtained using *n*-hexane. α-Pinene is a natural terpene hydrocarbon obtained from gum turpentine, a kind of essential oil distilled from pine gum. Gum turpentine, a renewable resource, has become a very important material as a solvent which is used to thin oil based paints and for producing varnishes. Gum turpentine obtained from pine forests, it used to produce high quality α-pinene, β-pinene, pine oil, terpineol and other terpenes. *p*-Cymene is an aromatic hydrocarbon that occurs widely in tree leaf oils [9]. It’s an important product and valuable intermediate in the chemical industry. Among others, it is used as a solvent for dyes and varnishes, as a heat transfer medium, as an additive in fragrances and musk perfumes, and as a masking odor for industrial products. To our knowledge, this work represents the first time that terpenes were used as solvent in extraction of oil-containing materials such as microalgae.

The relevant properties of those three terpenes as compared to *n*-hexane as solvent are listed in Table 1. The terpenes have similar molecular weights and structures to substitute *n*-hexane. Solubility parameters of solvents have been studied by means of Hansen Solubility Parameters (HSP) [10]. HSP were developed by Charles M. Hansen and provide a way to describe a solvent in terms of its non-polar, polar, and hydrogen bonding characteristics. The HSPs work on the idea of “like dissolves like” where one molecule is defined as being ‘like’ another if it bonds to itself in a similar way. The overall behavior of a solvent is characterized by three HSP parameters: δ_d_, the energy from dispersion bonds between molecules, δ_p_, the energy from dipolar intermolecular force between molecules and δ_h_, the energy from hydrogen bonds between molecules. *n*-Hexane and terpenes have similar values of the three descriptive terms; they likely behave similarly in practice. From this point of view, the terpenes are as effective as hexane to dissolve oils. However, considering their dielectric constants terpenes are slightly more polar and have more dissociating power than *n*-hexane. From the security point of view, terpenes have higher flash points than *n*-hexane, so they are less flammables and hazardous. The major drawback of using terpenes is their high viscosity and density, and also the higher energy consumption related to solvent recovery by evaporation due to their higher boiling point (155 °C and 176 °C) and higher enthalpies of vaporization (37–39 kJ/mol) compared to *n*-hexane (Bp = 69 °C, ΔHvap = 29.74 kJ/mol). 

**Table 1:** Relevant properties of *n*-hexane and terpenes.

Solvent	*n*-Hexane	*d*-Limonene	*α*-Pinene	*ρ*-Cymene
Chemical Structure	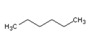	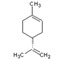	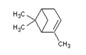	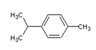
Molecular formula	C_6_H_14_	C_10_H_16_	C_10_H_16_	C_10_H_14_
Molecular weight (g·mol^−^^1^)	86.18	136.23	136.24	134.22
Density 25 °C (g·cm^−^^3^)	0.675	0.834	0.879	0.861
Flash point (°C)	−23	48.3	32	47.2
Boiling point (°C)	69	176	155	176
Viscosity, 25 °C (Cp)	0.31	0.83	1.32	0.83
Enthalpy of vaporization (kJ·mol^−1^)	29.74	39.49	37.83	39.34
Surface tension, 25 °C (dyne·cm^−^^1^)	20.3	25.8	25.3	28.5
Dielectric constant, 20 °C	1.87	2.44	2.58	2.34
Hansen Solubility Parameters δ^a^ (cal^1/2^·cm^−3/2^)	7.3	8	7.9	8.1
δ_d_	7.3	8	7.6	8.1
δ_p_	0	0.1	2.1	0.3
δ_h_	0	0.1	0	0

**Figure 1:**
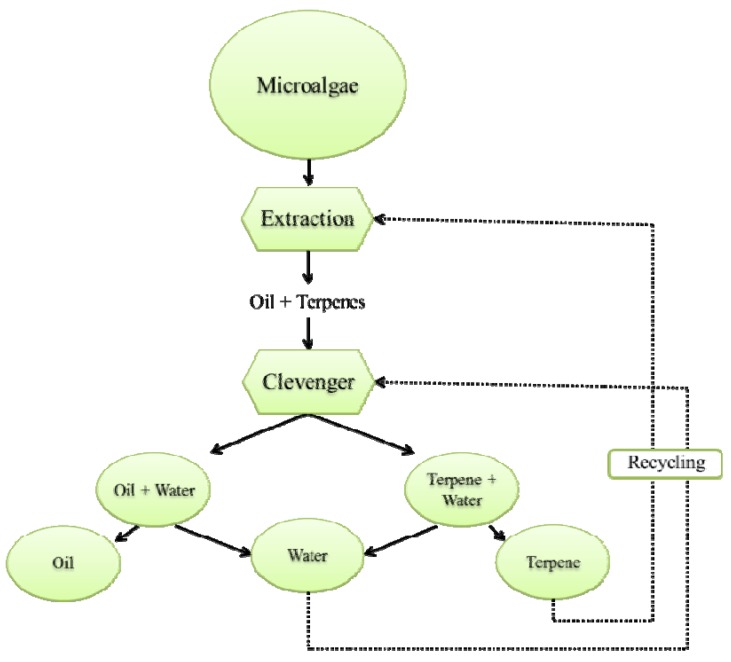
Extraction procedure using terpene solvents.

In order to resolve this problem of evaporation (energy and temperature), it is important to know how these bio-solvents have been extracted from the plant matrix. Terpenes are the primary constituents of the essential oils of many types of plants and flowers which are commonly extracted from their matrix by using water hetero-azeotropic distillation. The Clevenger apparatus has been used for decades in hydrodistillation in order to extract and measure essential oils contained in plants [11]. This process allows the extraction of compounds at low temperature, about 97–98 °C at atmospheric pressure and less if reduced pressure is applied, as compared to the high boiling point of terpenes contained in essential oils (150 to 300 °C). Based on this fact this way can be used for the recovery of terpenes such as *d*-limonene, *α*-pinene and *para*-cymene, resulting from the extraction step of oil from microalgae (Figure 1).

The aim of the study was to evaluate the possible extraction of oils from microalgae using terpenes as alternative solvents to *n*-hexane. Extraction step of oils from microalgae was thus investigated using Soxhlet extraction and the step of elimination of the solvent from the medium was carried out using Clevenger distillation. Extracted oils were then compared with oils obtained with *n*-hexane in terms of crude extract (quantitative results) and fatty acid composition (qualitative comparison).

## 2. Results and Discussion

Soxhlet extraction is recognized by the Association of Analytical Chemists (AOAC) as the standard method for crude fat analysis. Fat is extracted through repeated washing, or percolation, with an organic solvent under reflux in special glassware. Lipid extraction by Soxhlet is usually performed with apolar solvents such as *n*-hexane. Since *n*-hexane is considered to be a hazardous air pollutant, interest in alternatives to *n*-hexane as an extraction solvent has been stimulated. As can be seen in Figure 1, *n*-hexane has been substituated by alternative solvents obtained from renewable feedstocks such as *d*-limonene, α-pinene and *para*-cymene.

### 2.1. Extraction Kinetics

**Figure 2:**
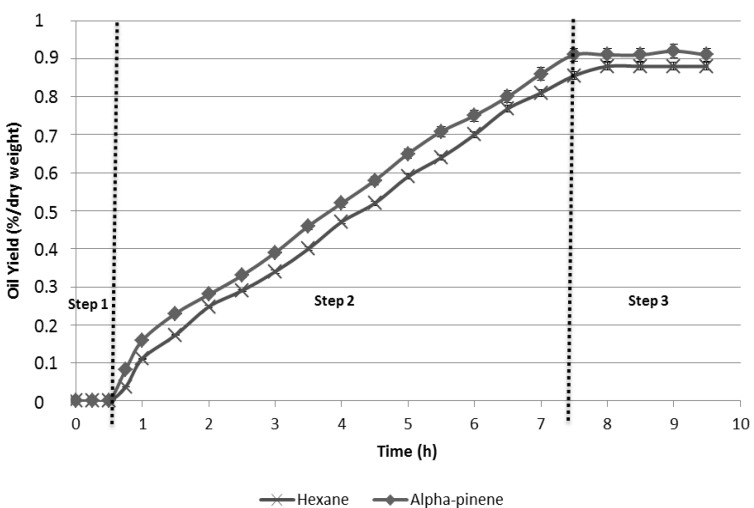
Extraction kinetics.

The variations of the extraction yields according to the extraction time are shown in Figure 2. The yields of oil extracted from microalgae with two different solvent are respectively 0.88% and 0.91% for *n*-hexane and α-pinene (*d*-limonene and *para*-cymene give the same trends). As is shown in Figure 2, the kinetic profiles and extraction rates for both solvents were similar. Three phases are observed: Step 1 represents the heating phase from room temperature to the boiling point of the solvent, Step 2 is represented by an increasing line which characterizes the diffusion of the oil from microalgae towards the external medium, and Step 3 corresponds to a horizontal line which marks the end of the extraction process.

### 2.2. Oil Extraction Yields from the Different Solvents

Extracted oils obtained using different solvents were compared in terms of qualitative determination. Yields of crude extract were thus checked and compared for all solvents using gravimetric determination and the method of Bligh and Dyer [12]. These results are listed in Table 2. The total lipids extraction (membranes and reserves lipids) was performed by the Bligh and Dyer method, which is the reference method for marine lipid extraction, and therefore is considered as 100% yield. The microalgae used contain a total yield of 3.36 g per 100 g of dry weight biomass. As can be seen in this table, extracted mass of crude oil was higher using terpenes than *n*-hexane. This effect has already been noted by Liu and Mamidipally [6], and might be due to the slightly more polar nature of terpenes compared with *n*-hexane. As a consequence more compounds can be extracted from the matrix. A higher dissolving ability of terpenes for polar lipids might also be pointed out by the higher temperature used to boil this solvent which can produce a lower viscosity of the analytes in the matrix and, in consequence, a better diffusion rate of the solute from the solid phase to the solvent.

**Table 2:** Fatty acid composition of lipids extracted from *C. vulgaris*.

Fatty Acids	Extracted amounts of fatty acids (%)				
	Bligh and Dyer	*n*-hexane	*d*-limonene	*α*-pinene	*ρ*-cymene
C14	1.29 ± 0.04	1.83 ± 0.11	0.98 ± 0.31	1.38 ± 0.06	1.43 ± 0.08
C16:0	26.63 ± 0.23	25.99 ± 0.52	27.62 ± 0.97	27.53 ± 0.32	28.91 ± 1.10
C16:1	4.93 ± 0.32	2.03 ± 0.09	4.95 ± 0.66	3.02 ± 0.21	4.47 ± 0.06
C16:2	1.37 ± 0.10	0.70 ± 0.03	2.83 ± 0.30	1.49 ± 0.17	0.92 ± 0.13
C16:3	3.27 ± 0.18	1.65 ± 0.48	2.35 ± 1.67	1.26 ± 0.18	1.39 ± 0.13
C16:4	9.89 ± 0.14	4.95 ± 1.78	6.64 ± 1.44	5.08 ± 0.16	3.36 ± 0.13
C18:0	1.67 ± 0.31	4.44 ± 0.08	6.37 ± 1.00	4.58 ± 0.11	3.92 ± 0.21
C18:1	17.79 ± 0.20	35.91 ± 0.07	23.97 ± 0.50	33.04 ± 0.10	36.70 ± 0.59
C18:2	6.99 ± 0.36	7.32 ± 0.24	5.87 ± 0.61	7.37 ± 0.31	5.49 ± 0.35
C18:3	23.48 ± 0.09	12.67 ± 0.10	14.55 ± 0.20	13.54 ± 0.07	12.33 ± 0.24
C18:4	2.50 ± 0.10	2.13 ± 0.09	3.64 ± 0.29	1.36 ± 0.08	0.66 ± 0.10
C20:0	0.20 ± 0.06	0.40 ± 0.04	0.23 ± 0.09	0.35 ± 0.08	0.41 ± 0.06
∑SFAs	29.7922.7247.49	32.6537.9429.41	35.228.935.9	33.8336.0630.11	34.6841.1724.15
∑MUFAs					
∑PUFAs					
**Oil Yield [%]**	**3.36 ± 0.02**	**0.88 ± 0.01**	**1.29 ± 0.01**	**0.91 ± 0.05**	**1.52 ± 0.03**

### 2.3. Oil Quality Comparison Results

The fatty acid composition of lipids extracted from *C. vulgaris* using terpenes and *n*-hexane as solvent were determined using GC analysis (Table 2). The main fatty acids identified in the oil fractions were 16:0, 18:1 and 18:2, with lower percentages of 14:0, 16:2, 16:3, and 20:0. Regarding the quantity and quality of fatty acids, there was no real variation in the lipid profile when extracted by *d*-limonene, α-pinene, *para*-cymene or *n*-hexane. The fatty acid present in largest amount was oleic acid (18:1), ranging between 23.97% and 36.70%. The presence of palmitic acid (16:0) was detected between 26.00 and 28.91% in the samples. This result was consistent with that obtained by Petkov and Garcia [13], 18:2 was observed in 25–56% in *C. vulgaris* biomass. In this work were also identified 18:2 and 18:3 polyunsaturated fatty acids. Similar amounts of polyunsaturated fatty acids in *C. vulgaris* were also observed by other authors [14]. The fatty acid 14:0 was present in all samples in small amounts, ranging from 0.98 to 1.83%. According to the literature the percentage of 14:0 in freshwater microalgae does not exceed 1% [13]. The unsaturated and saturated fatty acids content of *C. vulgaris* were 67.35% and 32.65%, respectively.

In particular, lipids with high content of unsaturated fatty acids have been reported to have a reasonable balance of fuel properties [15]. The chain length of fatty acids was between C14 and C20 in this study. In a previous report [16], fatty acids with chain length C14–C22 were recognized as the most common fatty acids contained in biodiesel. Therefore, fatty acids from *C. vulgaris* were suitable for the production of good quality biodiesel. It can be said that the proportion of the different fatty acids as well as the proportion of SFAs, PUFAs, or MUFAs has not been affected by the unusual conditions used in our experiment, in other words, the use of terpenes as solvent does not introduce extraneous effects and/or artifacts in the composition of the extracted oils.

### 2.4. Microscopic Observations

**Figure 3:**
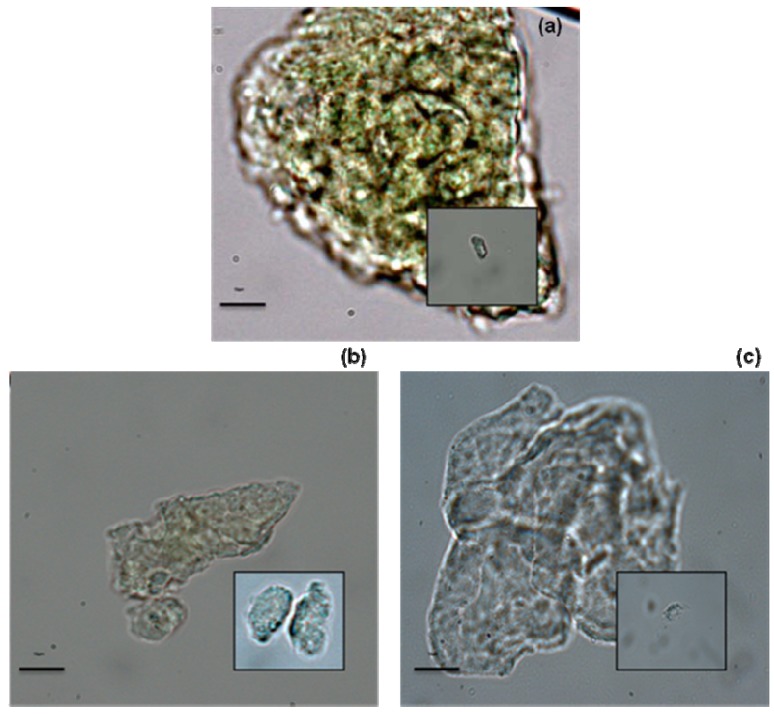
Microscopic observations of *Chlorella vugaris* (**a**) native microalgae (without extraction); (**b**) *n*-hexane extraction; (**c**) terpenes extraction.

Microalgae were observed by microscopic after extraction as shown in Figure 3; microscopic observations revealed a morphological heterogeneity, these differences could be due to the use of various solvents. Microalgae without extraction have an intact parietal system and cellular content. For *n*-hexane and terpenes extraction, the parietal system has been damaged and the cellular content was emptied including the chlorophyll according to Figure 3b,c. We can observe a stronger degradation of the membrane for extraction with terpenes; where is possible to see free cell organelles.

## 3. Experimental

### 3.1. Chemical and Reagents

Solvents used during extraction experiments (*d-*limonene, *α*-pinene, *para*-cymene or *n*-hexane) were of analytical grade and were supplied by VWR International (Darmstadt, Germany). Methanol, sulfuric acid, BHT, toluene and NaCl used for the preparation of fatty acid derivatives were all of analytical grade and were also purchased from VWR International. Various glasswares, Soxhlet apparatus and extraction thimbles used in extractions and fatty acid methyl ester preparations were supplied by Legallais (Montferrier-sur-Lez, France). 

### 3.2. Microalgae Cultivation and Harvesting

Commercial dry *Chlorella vulgaris* was obtained from Alphabiotech Company (Asserac, France). The microalgae were grown in raceway, with ambient air. After cultivation, the biomass was harvested by membrane filtration, and then centrifuged to obtain microalgae paste. All the microalgal paste was stored at −80 °C then freeze-drying.

### 3.3. Determination of the Total Lipid Content

The content of total lipids in the microalgae was determined by mixing with chloroform-methanol (1:2 v/v) using the Bligh and Dyer method [12]. The mixture was agitated during 15 min in an orbital shaker at room temperature. The lipid fraction was then separated and the solvent evaporated under a nitrogen stream. The lipids obtained were weighted and calculated (% dry weight) as standard for the following calculation.

### 3.4. Soxhlet Extraction

**Figure 4:**
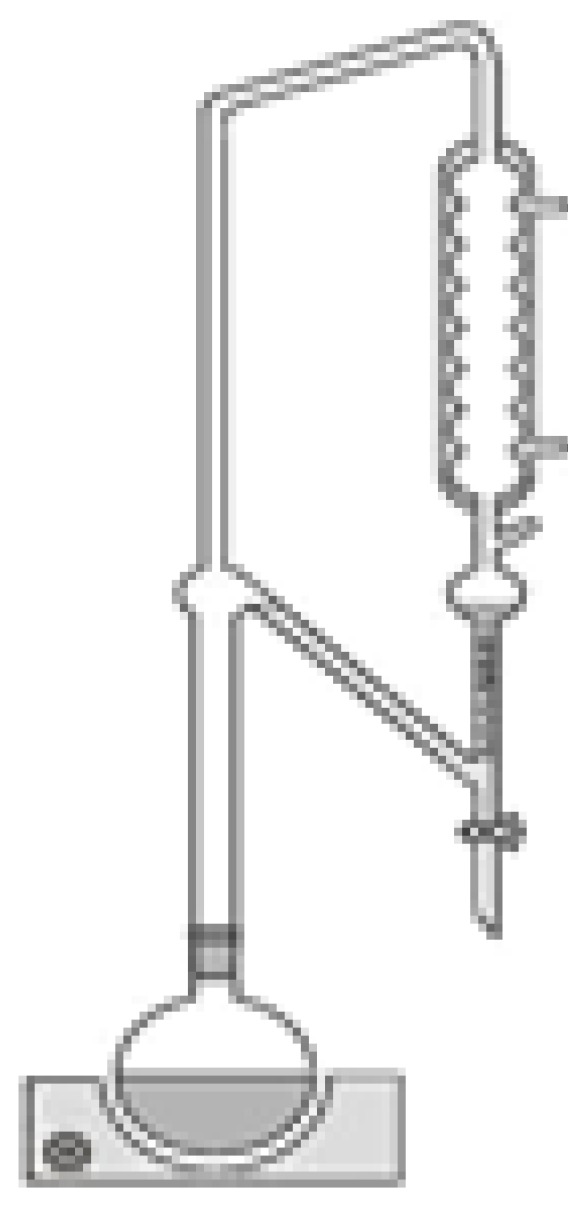
Clevenger distillation.

Oil was isolated from microalgae by means of Soxhlet extraction [17]. Dry microalgae (10 g) were weighed into a 30 mm × 80 mm cellulose thrimble (Macherey-Nagel) and placed in a Soxhlet apparatus. The oil in the microalgae was extracted for 8 h using 300 mL of four different solvents: *d*-limonene, *α*-pinene, *para-*cymene and *n*-hexane. In the case of hexane, solvent was removed; the weight of the algal oil was determined and used to calculate maximum recovery oil yields. Terpene solvent removal was performed by Clevenger distillation. To do this, the Soxhlet apparatus was replaced by Clevenger glassware on the distillation flask, as shown in Figure 4, but before distilled water was added to the mixture composed of extracted oil and terpene solvent. During azeotropic distillation of the binary water-terpene mixture, terpene solvent was eliminated from the distillation flask. Both used terpene solvent and extracted oil were recovered separately by phase separation. Terpene solvent was recovered from the water layer by phase separation in the “separating funnel” of the Clevenger glassware and the extracted oil was recovered from the water layer by phase separation in the distillation flask.

### 3.5. Preparation of Fatty Acid Methyl Ester Derivatives

The modified Morrison and Smith method was used to prepare fatty acid methyl ester (FAMEs) derivatives [18]. An acid catalysis was employed during derivatization procedures by using a defined amount of 5% methanolic sulfuric acid solution (1 mL) added to a specific amount of extracted oil. Internal standard used was glyceryl triheptadecanoate (C_54_H_104_O_6_). The mixture was then heated during 90 min at 85 °C. After, the flask was removed from heat and sodium chloride (1.5 mL, 0.9%) solution and n-hexane (1 mL) were added. The flask was stoppered and shaken vigorously during 30 s, then centrifuged at 4,000 rpm during 2 min. A small amount of the organic layer was removed and transferred in a vial before being injected directly in a gas chromatograph. 

### 3.6. Chromatographic Analysis of Fatty Acids

FAMEs were separated, quantified and identified by gas chromatography coupled with mass spectrometry (GC/MS). Analyses were performed by using a Shimadzu QP2010 (Kyoto, Japan) instrument equipped with a UB-Wax capillary column 30 m × 0.25 mm × 0.5 μm (Varian) and the velocity of the carrier gas (He) was at 35 cm/s. Injection of 2 µL of the various samples were carried out with a splitless mode and the injector temperature was set at 250 °C. Oven temperature was initially 50 °C for 1 min and then progressed at a rate of 20 °C/min from 50 °C to 190 °C and then increased from 190 °C to 230 °C at a rate of 2 °C/min. The temperature was then held at 230 °C for 15 min. The mass spectra were recorded at 3 scan/s from 50 to 380 a.m.u and the ionization mode was e.i at 70 eV. Identification of common fatty acids was a performed using the NIST’98 [US National Institute of Standards and Technology (NIST), Gaithersburg, MD, USA] mass spectral database.

### 3.7. Microscopic Observations

The impacts of the extracting methods on the structure of microalgae cells were examined using light microscopy. Microalgae residues were collected before (native microalgae) and after extractions and introduced in 70% ethanol at 6 °C. Microscopic observations were performed with a Leica DM 2000 Microscope (Wetzlar, Germany) equipped with DFC 30F digital camera (LAS software).

## 4. Conclusions

The aim of the present study was to investigate an alternative procedure for the determination of oils using terpenes as alternative solvents. This aim was achieved with good results in terms of gravimetric determination and fatty acid composition. It can be said that the proposed investigation is a valuable and effective method for oil determination in microalgae. However, it is important to note that high temperature of terpenes can have a positive impact on lipid yield. It would be interesting to test the effectiveness of hexane under pressure at 176 °C. 
